# Orthogonal Design and Microstructure Mechanism Analysis of Novel Bentonite Polymer Slurry in Pipe Jacking

**DOI:** 10.3390/polym15061461

**Published:** 2023-03-15

**Authors:** Jimin Liu, Xiangzhi Wang, Hua Cheng, Haixu Fan

**Affiliations:** 1 School of Civil Engineering and Architecture, Anhui University of Science and Technology, Huainan 232001, China; 2 Anhui Province Key Laboratory of Building Structure and Underground Engineering, Anhui Jianzhu University, Hefei 230009, China; 3 Engineering Research Center of Underground Mine Construction, Ministry of Education, Anhui University of Science and Technology, Huainan 232001, China

**Keywords:** grouting material, crosslinked polymers, optimal proportion, microstructure analysis, slurry pipe jacking, working mechanism

## Abstract

The selection of an appropriate slurry ingredient and its percentage ratio is a vital and necessary task for engineers in slurry pipe jacking operations. However, traditional bentonite grouting materials are difficult to degrade because of their single and non-biodegradable composition. Nowadays crosslinked polymers have been widely considered due to their excellent performance and application in engineering practices, which enlighten novel polymer slurry in pipe jacking. This study innovatively proposed using boric acid crosslinked polymers added into polyacrylamide bentonite slurry, which not only solves the shortcomings of traditional grouting materials but also meets the general working performance requirements. The new slurry’s funnel viscosity, filter loss, water dissociation ratio and dynamic shear were tested according to an orthogonal experiment. Single factor range analysis was conducted to identify the optimal mix proportion based on an orthogonal design, and the formation behavior of mineral crystals and microstructure characteristics were evaluated by X-ray diffraction and scanning electron microscopy respectively. According to the results, guar gum and borax form a dense boric acid crosslinked polymer through cross-linking reaction. The internal structure grew tighter and more continuous as the crosslinked polymer concentration grew. It improved the anti-permeability plugging action and viscosity of slurries by 36.1~94.3%. The optimal proportions of sodium bentonite, guar gum, polyacrylamide, borax, and water were 10%, 0.2%, 0.25%, 0.1%, and 89.45% respectively. All these works indicated that the improvement of slurry composition by using boric acid crosslinked polymers was feasible.

## 1. Introduction

As one of the most popular methods for utilizing underground spaces, pipe jacking has been widely used in the area of oil and gas, water supply, sewage, communication and electricity pipelines, and pipe-roof projects. It employs hydraulic jacks to push specially made pipes through the ground behind a jacking machine, from a drive shaft to a reception shaft. In pipe jacking engineering, the synchronous grouting method is extensively employed in the construction of municipal and river-crossing pipeline engineering all over the world. It is also called slurry pipe jacking [1,2,3,4]. The pipeline segments are lifted smoothly and effectively thanks to the thixotropy properties of the synchronous grouting slurry. The soils around the segments are well-protected as well [5,6,7,8]. On the one hand, the slurry becomes a gelatinous liquid with poor viscosity and strong fluidity when it is stirred, vibrated, or pumped. It provides necessary friction resistance around the pipe segments. On the other hand, the slurry turns into a gel with increasing viscosity when it stops stirring, vibrating, or pumping, it fills the over-excavation area effectively, stabilizes the surrounding strata and reduces the settlement deformation. Therefore, the key to slurry pipe jacking construction is to prepare a suitable thixotropy slurry.

At present, the thixotropy slurry used in pipe jacking engineering mostly consists of water and clay or bentonite. Additives (mainly polymers) are used under some circumstances to enhance their properties. Compared with calcium bentonite, sodium bentonite slurry has greater gelling and solidification properties after standing, as well as better fluidity following thixotropy. So, sodium bentonite is more popular in pipe jacking engineering [9,10]. The additives added to the sodium-based bentonite slurry are generally polyacrylamide, carboxymethyl cellulose, polyanionic cellulose, etc. [11,12,13,14]. But it is revealed from further research that the slurry which consists of clay, bentonite, water and carboxymethyl cellulose makes slurry difficult to degrade and pollutes the environment [15,16]. The study on more efficient and less toxic alternatives of polymers makes sense, more reasonable components and ratio design should be studied in depth.

Over the past few decades, advanced synthesis techniques allow for the synthesis of polymers with controlled molecular architecture and diverse functionality, further promoting the potential applications of polymer-based materials in the lubrication field. Among them, the crosslinked polymer has attracted many researchers and has been an avail ingredient to improve the working performance in the industry [17,18,19,20,21,22]. Through the crosslinking reaction between polymer and borate, it will cross-link to form a gel structure with good viscoelasticity. When this gel is sheared and heated, the B-O bond at the crosslinking point is broken, which reduces the viscosity of the fluid. When the shearing and heating effects are removed, they can be crosslinked again, thereby restoring the viscosity. Furthermore, it is clean, cheap, compatible with resin-coated proppant and environmentally friendly. The borate-crosslinked polymer can formulate a low-permeability filter cake. It can protect the stratum from collapsing and stabilize the soils together with other additives. All these characteristics satisfy the working requirements of slurry in pipe jacking.

Therefore, we propose the idea of introducing boric acid crosslinked polymers into sodium bentonite slurry in pipe jacking engineering. In general, the crosslinked polymer generally included xanthan gum, guar gum, cellulose, and starch [23,24]. In this study, guar gum, which was one of the popular crosslinked polymers, was selected as the raw material. Besides, sodium borate was chosen as another raw material for the crosslinking reaction. They were added into polyacrylamide bentonite to form a new polymer bentonite slurry. As the slurry in pipe jacking was very similar to water-based drilling fluids in terms of function and composition, the preparation and performance evaluation often used the rheological performance index of drilling fluid [25,26,27]. This method was also applicable in this novel polymer bentonite slurry. A series of experimental analyses on the macroscopic and microscopic properties were conducted in this study. Four macroscopic indicators were used to select the optimal slurry component proportion, including funnel viscosity (FV), filter loss (FL), water dissociation ratio (WDR), and dynamic shear (DS). Multi-components and multi-levels were introduced in the orthogonal design, and the optimization level range of each macroscopic indicator was determined by range analysis. The mineral formation behavior was analyzed by X-ray diffraction (XRD), and the characteristics of both the microstructure and polymerization reaction were analyzed by scanning electron microscopy (SEM).

## 2. Experimental Materials and Methods

### 2.1. Experimental Materials

#### 2.1.1. Raw Materials

**Figure 1:**
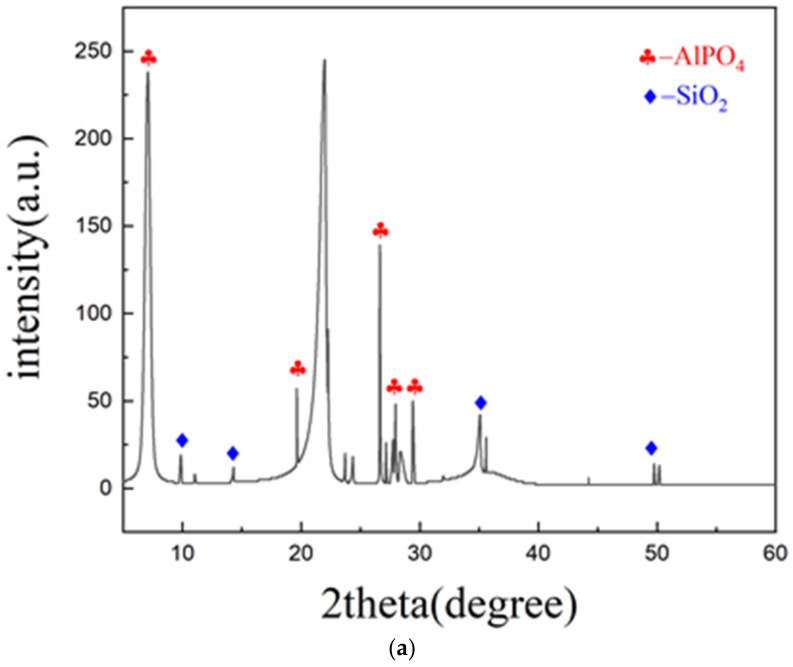

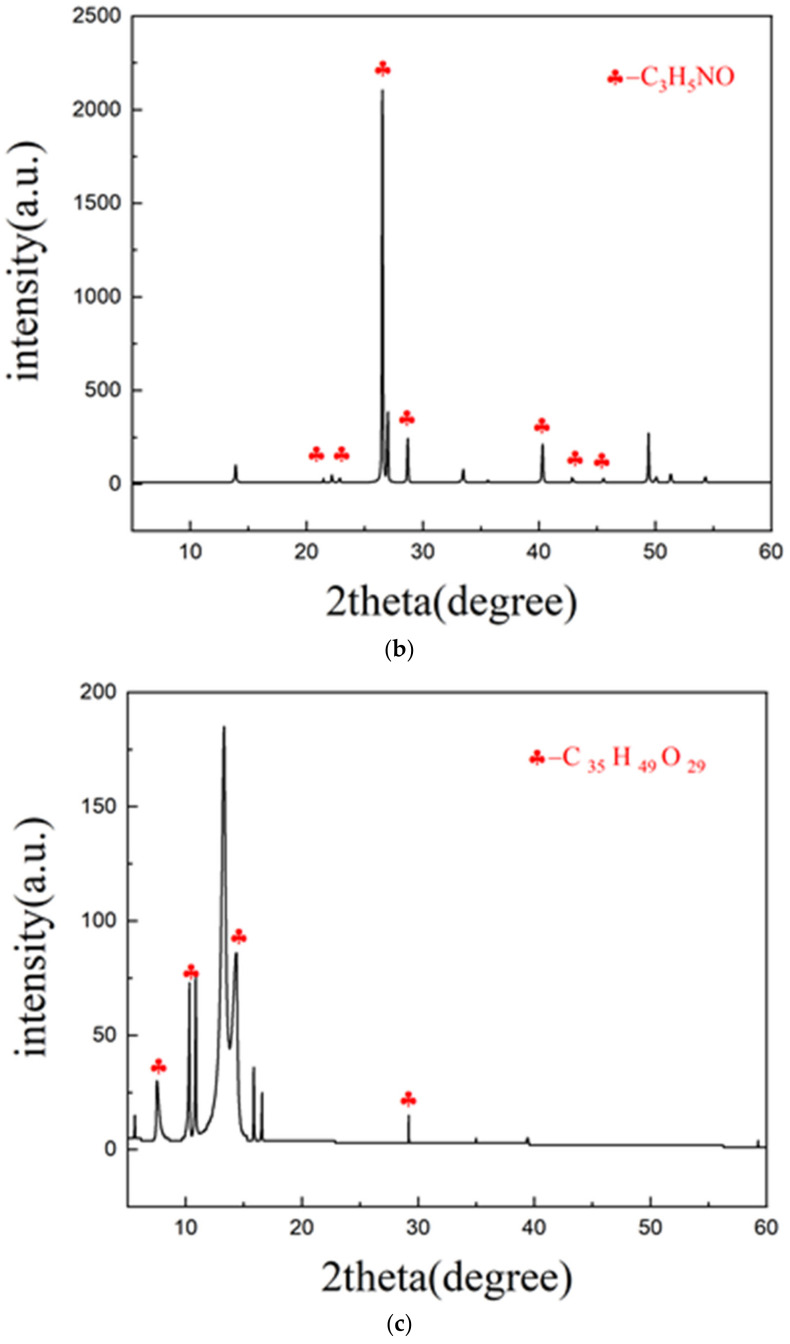

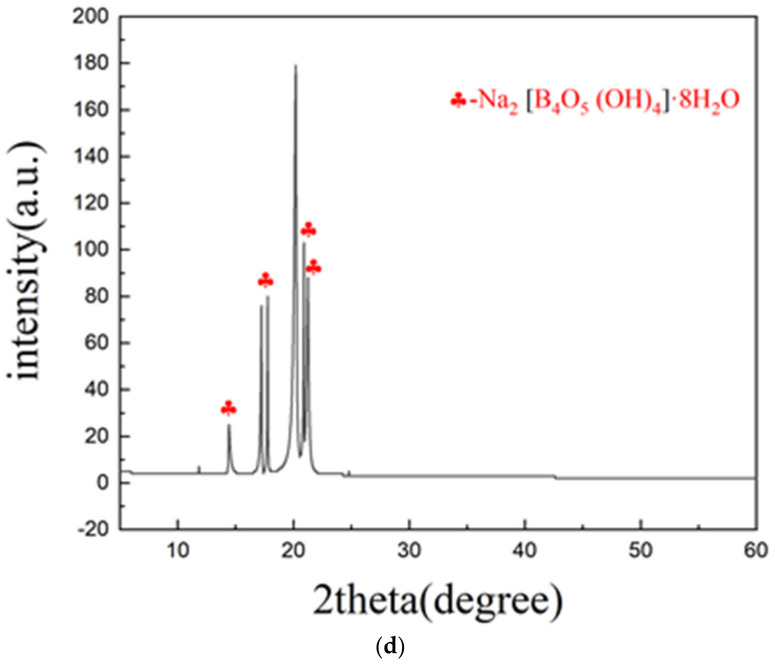
X-ray diffraction (XRD) pattern of all components. (**a**) Sodium bentonite. (**b**) Polyacrylamide. (**c**) Guar gum. (**d**) Borax.

This experiment used sodium bentonite, provided by the Hengxin filter material factory (Gongyi, China). Mineralogically, it containedabout 75% sodium montmorillonite, 21.5% Illisite-kaolinite, and small amounts of quartz and plagioclase. The mineralogy of the bentonite was measured using an X-ray fluorescence 1000 spectrometer (XRF) as shown in Figure 1a. Its main physical properties are shown in Table 1.

Polyacrylamide was purchased from Fuchen chemical reagent Co., Ltd. (Tianjin, China). Its purity was no less than 90%. The mineralogy of polyacrylamide was also measured using XRF, as shown in Figure 1b. Guar gum was produced by Shengyuan biological Co., Ltd. (Zhoukou, China). Its viscosity was 5600 mpa∙s, drying reduction was 12.1%, ash content was 0.42%, and acid insoluble matter was 2.78%. Its mineralogy isshown in Figure 1c. The XRF results showed the main chemical composition of Guar gum was C_35_H_49_O_29_.

Borax was made by the Dengfeng chemical reagent factory (Tianjin, China). Its purity was greater than 99.5% and other chemical compositions besides the main components were shown in Table 2. The mineralogy of borax was shown in Figure 1d. The XRF results showed the main chemical composition of borax was Na_2_[B_4_O_5_(OH)_4_].8H_2_O. The chemical composition of all components tested by X-ray fluorescence (XRF)spectrum analysis was summarily listed in Table 3.

#### 2.1.2. Preparation of Specimens

The preparation of specimens followed the steps below: weighed the above-mentioned raw materials in proportion firstly, added with the municipal tap water to comply with the design scheme, and mixed with a low-speed mixer at a speed of 550~600 rpm for 30 min in a plastic bucket. All components were used without further purification.

### 2.2. Macroscopic Experiment Methods

#### 2.2.1. Funnel Viscosity Test

FV was the most basic rheological parameter of slurries, and it was tested by using a funnel viscometer. After calibrating the instrument, held the funnel and blocked the flow outlet with my fingers, injected the slurry into the funnel through the screen. Maintaining the funnel’s upright position while moving the finger away and pressing the stopwatch. The funnel viscosity of slurries was the time required to fill 946 mL of drilling fluid.

#### 2.2.2. Filter Loss Test

An API filter tester (ZNS-2A, Shandong, China) was applied to obtain the FL of slurries. FL was the volume of the water filtered from the slurry sample after standing for 30 min under a pressure of 0.69 MPa. A tire pump and a pressure gauge were used as a device to maintain the pressure at a constant value.

#### 2.2.3. Water Dissociation Ratio Test

A set of 1000 mL measuring cylinders was employed to test the WDR of slurries in the laboratory. The prepared slurry was injected into the measuring cylinder. The WDR was defined as the ratio of the water volume separated from the original slurry after the slurry has been set for 24 h.

#### 2.2.4. Dynamic Shear Test

A six-speed rotational viscometer (ZNN-D6S, Shandong, China) was used to measure the DS of slurries. At 300 and 600 rpm of the viscometer, recorded the torsion grid number $\phi_{300}$ $\phi_{600}$ respectively by a rotational viscometer. The DS of slurries can be calculated using the formula below.

(1) $$DS=5\left(2\phi_{300}-\phi_{600}\right)$$

### 2.3. Microscopic Experiments Methods

A FlexSEM1000 scanning electron microscope with a 10 kV acceleration voltage (Hitachi, Japan) was used to analyze the microstructure of specimens. The bonding characteristics of polyacrylamide bentonite, the structural characteristics of crosslinked polymer products, and the effects of their mixture on the microstructure were observed under 1000× magnification.

## 3. Orthogonal Experiment and Analysis

### 3.1. Orthogonal Experiment Design

The orthogonal test is an efficient and reasonable test design method, which can study the multi-factor and multi-level distribution law of components and obtain the optimal mix proportion. In this orthogonal experiment, the contents of sodium, guar gum, polyacrylamide and borax were set as four influencing factors, and five levels were set for each factor. The orthogonal design table is shown in Table 4. According to the orthogonal table, 25 groups of experiments were organized, and four indicators were measured following its method. The results are listed in Table 5.

### 3.2. Range Analysis

According to the orthogonal test results, the range analysis is performed for each index of slurries. The main evaluation criteria are the calculated kj and Rj values. The analysis method begins with the average value of each index related to a certain level of the factor. Repeat this step for other levels of this factor and obtain kj values. The difference between the maximum and minimum kj values might be used to calculate the Rj value, which is the range value of each design factor. The roles of Rj values and kj values are to determine the order of priority of the factors on the influence degree of each index and to give the optimal level of each factor and the optimal combination. The results of the range analysis are shown in Table 6.

#### 3.2.1. Range of Funnel Viscosity

**Figure 2:**
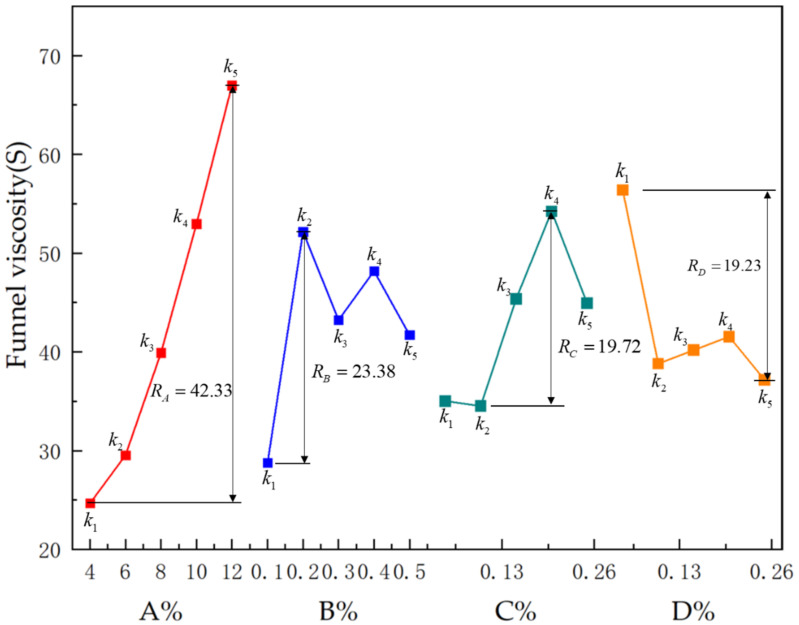
Effect of various factors on funnel viscosity.

Based on the statistical results in Table 6, all of the kj values of the level test results for each factor suggested that FV can be enhanced by using sodium bentonite, guar gum, and polyacrylamide. The Rj values of each factor were calculated based on the orthogonal test results, and the calculated values of the four components were 42.33, 23.38, 19.72, and 19.23 respectively. They are arranged $R_{A}>R_{B}>R_{D}>R_{C}$, indicating the effects of the factors on FV, and a more obvious representation is shown in Figure 2. FV was maximum at 12% sodium bentonite admixture and 0.2% polyacrylamide, 0.05% borax. As the long-distance transportation for slurry needs to have a certain resistance to deformation, the funnel viscosity value should not be too low. Given the need for sufficient FV and economic costs, the best possible combination is A5B2C4D1 or A4B2C4D1.

#### 3.2.2. Range of Filter Loss

**Figure 3:**
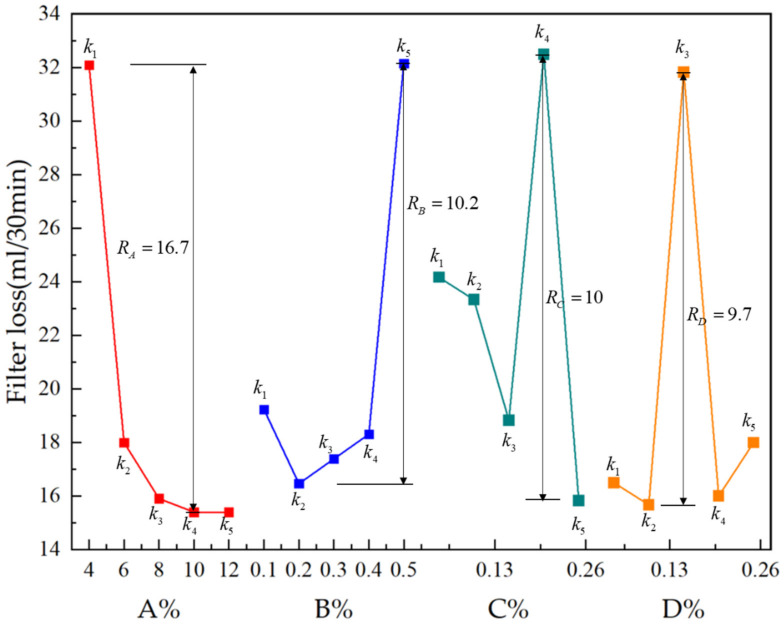
Effect of various factors on filter loss.

In line with the kj values variation of FL in Table 6 and the corresponding analysis in Figure 3, FL decreased when bentonite and polyacrylamide increased. FL was lowest when sodium bentonite content was 10%, guar gum content was 0.2%, polyacrylamide was 0.25% and the borax concentration was 0.1%. The Rj values of four components concerning FL were calculated as 16.7, 10.2, 10, and 9.7 respectively based on the orthogonal test results. Each factor’s range values were shown in alphabetical order $R_{A}>R_{B}>R_{C}>R_{D}$. Given that the slurry needs to play a certain role in reducing seepage and plugging leaks, the amount of water penetrating the soil should not be too much, so FL should not be too large. Combined with the above range analysis conclusions, A4B2C5D2 or A5B2C5D2 is the ideal combination when FL is considered.

#### 3.2.3. Range of Water Dissociation Ratio

**Figure 4:**
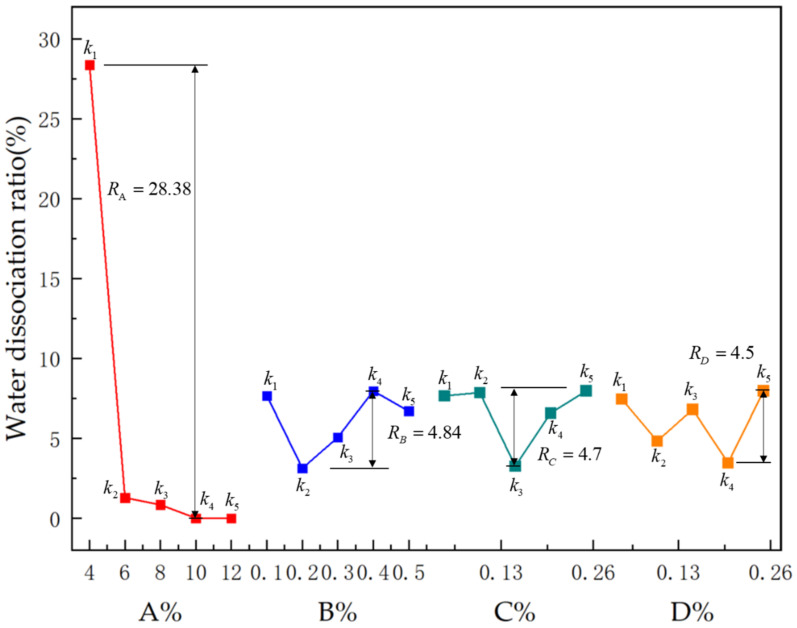
Effect of various factors on water dissociation ratio.

Per the kj values variation of WDR in Table 6 and Figure 4, the change between WDR and levels associated with each factor was not obvious except for sodium bentonite. The Rj values of four components concerning WDR were calculated as 28.38, 4.84, 4.7, and 4.5 respectively. Each factor’s range values were ranked as $R_{A}>R_{B}>R_{C}>R_{D}$. WDR was lowest when sodium bentonite content reached 10%, guar gum content was 0.2%, polyacrylamide content was 0.15% and borax concentration was 0.1%. When sodium bentonite content was 10%, the amount of polyacrylamide and borax had little effect on WDR. Considering that the general precipitation rate of WDR of pipe jacking slurry needs to be controlled within 10%, the proper combination considering WDR is determined as A4B2C3D4.

#### 3.2.4. Range of Dynamic Shear

**Figure 5:**
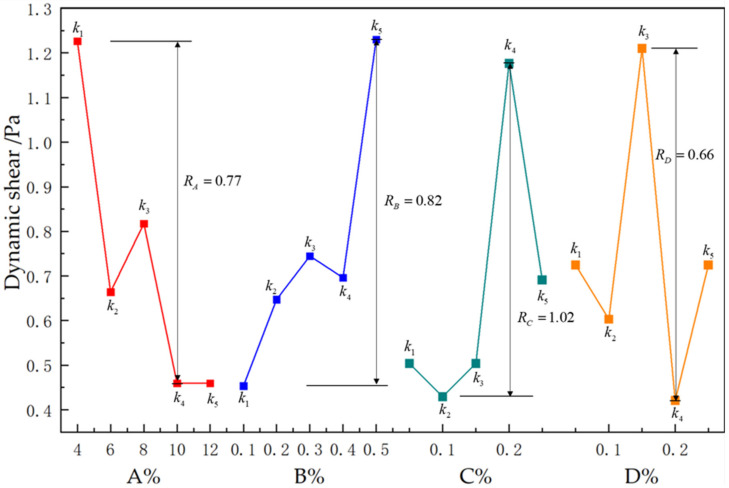
Effect of various factors on dynamic shear.

According to the kj values variation of DS in Table 6 and Figure 5, the change regularity with each factor at different levels was not apparent. The Rj values of four components concerning DS were calculated as 0.77, 0.82, 1.02, and 0.66 respectively. Each factor’s range values were ranked as $R_{C}>R_{B}>R_{A}>R_{D}$. DS was highest when the sodium bentonite content reached 4%, guar gum content was 0.5%, polyacrylamide content was 0.2% and the borax concentration was 0.15%. Since the dynamic shear force is the interaction force between clay particles and polymer molecules, its magnitude reflects the strength of the ability to form a spatial grid structure. Too high DS will cause an increase in pumping resistance and increase the resistance when restarting after stopping drilling. Too low DS will cause the slurry to lack suspension ability. Generally, it is controlled between 0.5~15 Pa. Considering the requirements on DS, A4B2C5D2 is the rational combination.

### 3.3. Determination of Optimized Slurry

Combined with the foregoing analysis, an increase in sodium bentonite increases FV and reduces FL and DS. It is the most important component of slurry. Increasing polyacrylamide and guar gum raises FV and DS. Borax reduces FV and DS. Comprehensively considering the influence of each component on the performances and the general working requirements, the optimal composition of the slurry is obtained as A4B2C5D2.

## 4. Microscopic Results and Analysis

**Figure 6:**
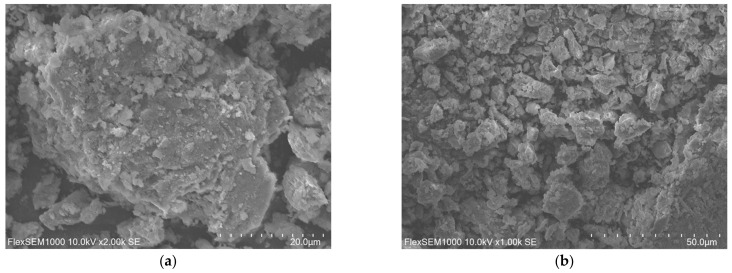

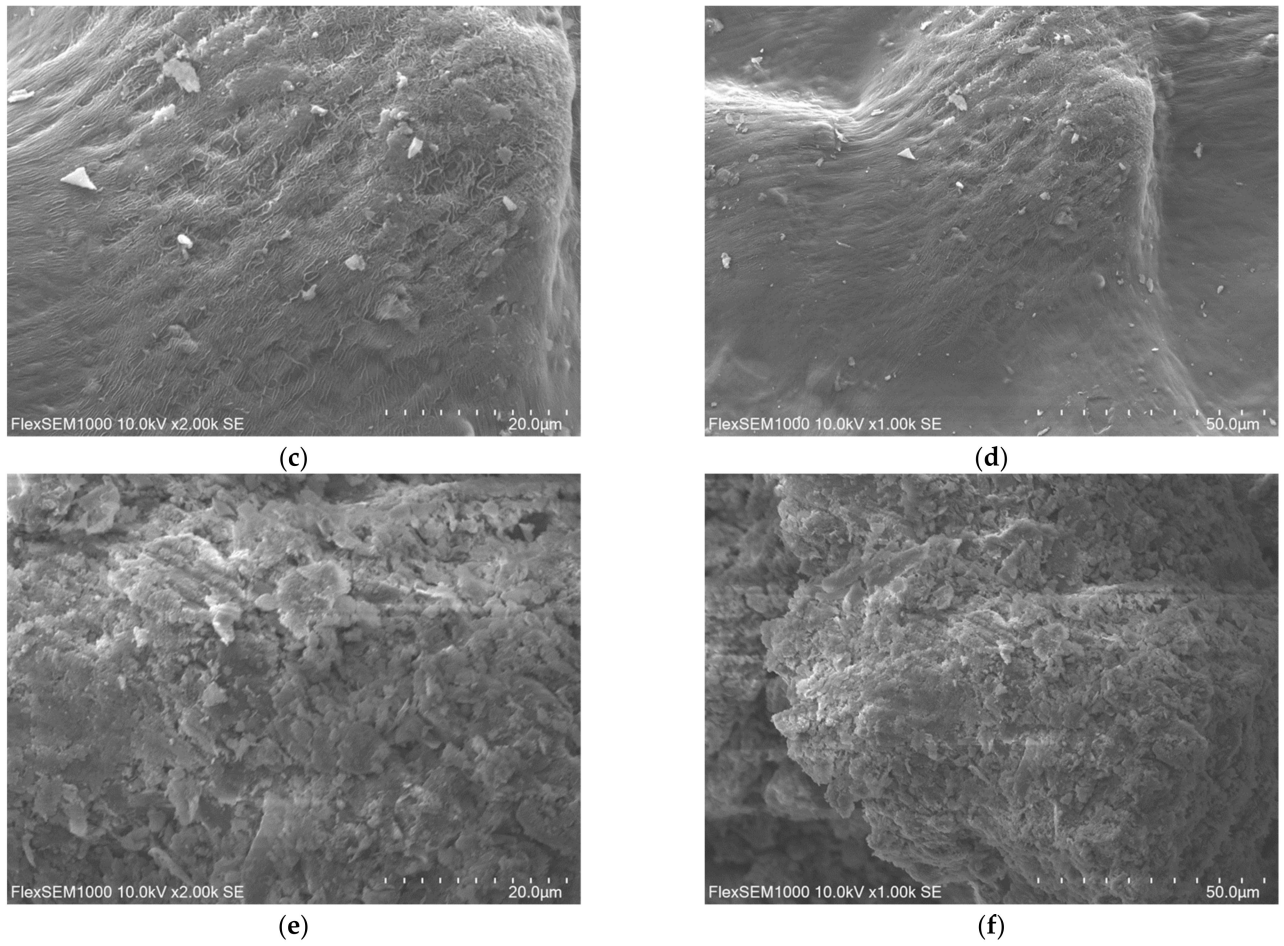
SEM analysis of specimens. (**a**) polyacrylamide bentonite specimen at 20 μm. (**b**) polyacrylamide bentonite specimen at 50 μm. (**c**) guar gum- borax crosslinked polymer specimen at 20 μm. (**d**) guar gum- borax crosslinked polymer specimen at 50 μm. (**e**) novel polymer bentonite specimen at 20 μm. (**f**) novel polymer bentonite specimen at 50 μm.

Three specimens of polyacrylamide bentonite, guar gum-borax crosslinked polymer and their composition were prepared according to optimal composition and magnified under two different times. Figure 6 showed their SEM results.

### 4.1. Microstructure of Polyacrylamide Bentonite

The microstructure of polyacrylamide bentonite was completely composed of fine particles, and no large blocks or gel structures were formed which was shown in Figure 6a,b. This microscopic performance was consistent with the conclusion in the literature [11,14]. The reason for this microscopic performance was that long chain molecules of polyacrylamide adhere to the surface of bentonite particles, which made the bentonite particles embedded in the mesh, partially blocking the internal pores. But there was still a certain amount of pores, cracks, and other defects distributed. The inner structure was porous and discontinuous.

### 4.2. Microstructure of Crosslinked Polymer

When guar gum interacted with borax, a crosslinking reaction occurred. The mechanism of the crosslinking reaction has been discussed by many researchers and applied in industries [28,29]. After being dissolved in water, the solution of borax was alkaline. In an alkaline environment, borax undergoes a hydrolysis reaction to generate negatively charged borate ions. However, some ortho cis hydroxyl groups in guar gum can react with these negatively charged borate ions to form complexes. The chemical reaction formula is shown in Formulas (2)~(4), Where R was the guar gum molecular chain group in Formula (4).

(2) $$Na_{2}B_{4}O_{7}+7H_{2}O↔2Na^{+}+2B{\left(OH\right)}_{3}+2B{\left(OH\right)}_{4}^{-}$$

(3) $$B{\left(OH\right)}_{3}+H_{2}O↔B{\left(OH\right)}_{4}^{-}+H^{+}$$

(4) $$B{\left(OH\right)}_{4}^{-}+2_{OH}^{OH}\rangle R↔R\langle_{O}^{O}\rangle B\langle_{O}^{O}\rangle R+4H_{2}O$$

**Figure 7:**
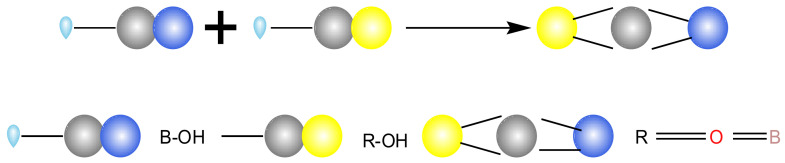
Crosslinking reaction process.

When the pH value increased, the ions were neutralized and the balance moved to the right, which was the direction of generation. It reacted with the orthosis hydroxyl group on the guar gum polymer chain and then cross-linked to form a colloid. The main chemical bond B-O bond in borax combined with free water to form B-OH. B-OH reacted with R-OH of guar gum to form a network structure, which increased the impermeability of the slurry. The reaction process was shown in Figure 7.

Therefore, the cross-linked polymer produced by the cross-linking reaction appeared in the form of long-chain molecules. Under the microscope of different magnifications, the long-chain molecule has a dense structure, no blocks inside and is arranged consecutively and orderly, as shown in Figure 6c,d.

### 4.3. Microstructure of Novel Polymer Bentonite

When the crosslinked polymer was added to polyacrylamide bentonite, the structure became dense, as shown in Figure 6e,f. The incorporation of crosslinked polymer improved the microstructure. Compared with polyacrylamide bentonite specimens, internal pores were reduced significantly. There were no obvious blocks inside, the structure became tighter and more continuous. It can be inferred that with the addition of crosslinked polymer, the internal structure changed significantly, and the properties of plugging and anti-penetration enhanced up to a point.

The above-mentioned changes in slurry microstructure after adding crosslinked polymer greatly proved the function of crosslinked polymer in novel bentonite slurry. With the addition of crosslinked polymer, a novel bentonite polymer slurry was prepared. This finding can be used in slurry pipe jacking engineering to satisfy the working requirement of plugging and anti-penetration ability in sandy formations.

**Figure 8:**
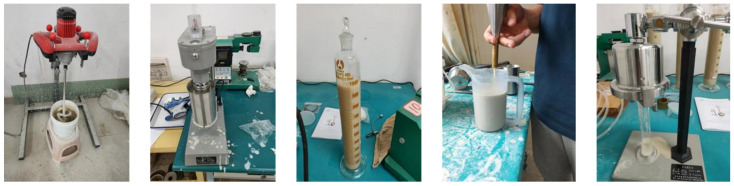
Indoor test preparation process.

The laboratory grouting model experiment were shown in Figure 8. In general, the method of slurry grouting was the combination of synchronous grouting from the tail of the micro tunnel boring machine and grouting from the precast holes in pipe segments along pipelines or only adopted one way. Literature [3] has described the actual grouting process in slurry pipe jacking engineering in detail. It was the same synchronous grouting technology as the novel bentonite polymer slurry in this paper.

**Figure 9:**
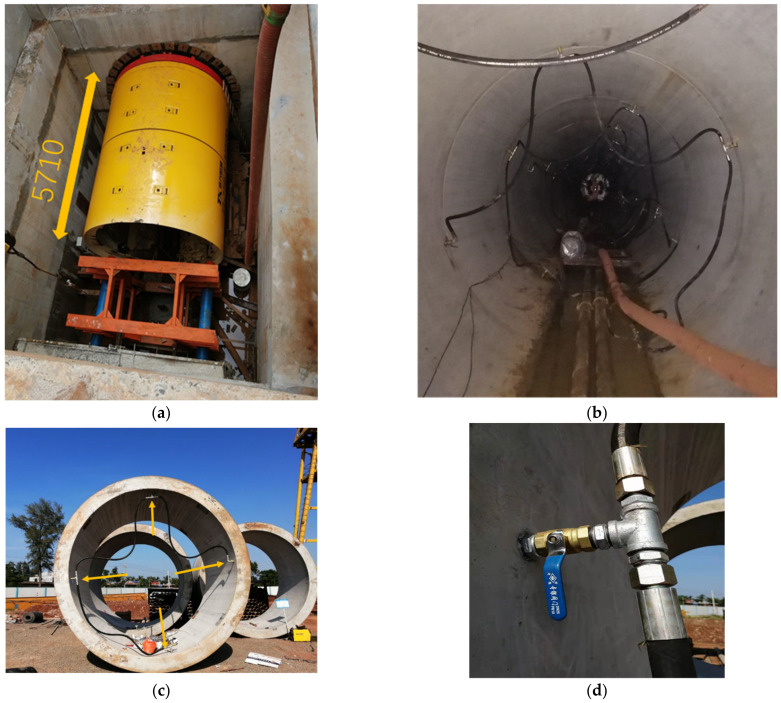
Site view of slurry grouting system during pipe jacking [3]. (**a**) Head of the micro tunnel boring machine. (**b**) Synchronous grouting on the pipeline segment. (**c**) Prefabricated grouting hole on each segment and the branch grouting pipe. (**d**) Ball valve controller on concrete pipe.

The whole slurry grouting system during pipe jacking was shown in Figure 9. To execute the slurry synchronous grouting of pipelines, a pipeline system injected slurry into the prefabricated grouting holes of the segment and the gap at the end of the shield machine. On each section, four grouting holes were reserved, which were located in the upper, lower, left, and right directions of the pipeline, respectively. Each grouting hole was individually controlled by a DN25 ball valve, and the grouting hole was connected to the main grouting pipe by a DN25 rubber pipe. The main grouting pipe was DN50 galvanized steel pipe, and the branch pipe was DN25 pressure-resistant rubber tubing, with a stainless-steel diaphragm pressure gauge positioned every 100 m to manage the grouting pressure. The whole grouting process was controlled and supervised by stainless steel pressure gauge.

## 5. Conclusions

In this study, a novel polymer bentonite slurry was prepared by different mix proportions. XRF was used to determine the mineral crystal characteristics of various components. The influence of different proportions of these components on the development of funnel viscosity, filter loss, water dissociation ratio and dynamic shear was analyzed. Finally, the microstructure and morphological characteristics were analyzed by SEM. The results are summarized as follows:

1. The influence of the component content on slurry properties has a certain regularity. An increase in sodium bentonite enhances funnel viscosity and reduces filter loss and dynamic shear. Polyacrylamide and guar gum raises funnel viscosity and dynamic shear. Borax reduces funnel viscosity and dynamic shear. When considering the influence of FL and WDR, the calculated range value was 16.7, 10.2, 10, 9.7 and 28.38, 4.84, 4.7, 4.5 respectively, which indicated the four components arranged as sodium bentonite > guar gum > polyacrylamide > borax. The order changed to sodium bentonite > guar gum > borax > polyacrylamide when considering FV and listed as polyacrylamide > guar gum > sodium bentonite > borax when considering DS.
2. Orthogonal test results indicated that the content of each component must take the appropriate intermediate value to best satisfy the comprehensive performance requirements in slurry pipe jacking engineering. Comprehensively considering the macro index of slurry and the general engineering needs, the optimized formula was sodium bentonite: guar gum: polyacrylamide: borax: water = 10:0.2:0.25:0.1:89.45.
3. SEM analysis showed that the incorporation of guar gum and borax continuously enhance the crosslink reaction efficiency, and the addition of crosslinked polymer considerably reduced the number of cracks and pores in polyacrylamide bentonite slurry. With the addition of crosslinked polymer to polyacrylamide bentonite slurry, both the microstructure and anti-penetration properties were continuously improved.
4. The admixture of crosslinked polymer and polyacrylamide bentonite can work together synergistically. This novel polymer bentonite slurry can meet the general performance requirements in pipe jacking engineering. It was prepared in the laboratory and can be applied in slurry pipe jacking engineering through synchronous grouting technology. but its adaptability and feasibility to different formations need to be furtherly tested by the projects.
5. On the whole, this novel bentonite polymer slurry made up of sodium bentonite, polyacrylamide, guar gum and borax can overcome the shortcoming of traditional polymer slurry. Both the macroscopic and microscopic properties test indicated it can satisfy the general performance requirements for pipe jacking engineering. Its superiority will be verified in future projects.

## Figures and Tables

**Table 1:** Index properties of the sodium bentonite.

Index	pH Value	Expansion Index (mL/g)	Glue Value (mL/15 g)	Water Absorption (%)	Moisture (%)	Wet Compressive Strength (MPa)
Sample	7.1	35	100.2	295	6	1.35

**Table 2:** Other chemical compositions besides the main components of borax.

Index	Hydrochloric Acid Insoluble Matter (%)	Chloride (%)	Sulfate (%)	Phosphate (%)	Calcium (%)	Iron (%)	Copper (%)
Sample	<0.005	<0.002	<0.01	<0.002	<0.005	<0.0003	<0.001

**Table 3:** Chemical composition of all components.

Components	Chemical Composition/%														
	SiO_2_	(C_3_H_5_NO)n	Acid Non-Soluble Substance	Na_2_ [B_4_O_5_ (OH)_4_]·8H_2_O	Al_2_O_3_	Fe_2_O_3_	Loss On Ignition	CaO	K_2_O	MgO	Na_2_O	TiO_2_	MnO	P_2_O_5_	Others
Sodium bentonite	68.08				16.07	4.31	3.47	2.45	2.11	1.36	1.33	0.67	0.1	0.05	
Polyacrylamide		99													1
Guar gum			2.78												97.22
Borax			0.005	99.5											0.495

**Table 4:** Factors and levels of the orthogonal test.

Levels	Factors			
	Sodium Bentonite Contents (%) A	Guar Gum Contents (%) B	Polyacrylamide Contents (%) C	Borax Contents (%) D
Level 1	4	0.1	0.05	0.05
Level 2	6	0.2	0.1	0.1
Level 3	8	0.3	0.15	0.15
Level 4	10	0.4	0.2	0.2
Level 5	12	0.5	0.25	0.25

**Table 5:** Results of the orthogonal test.

No.	A/%	B/%	C/%	D/%	Funnel Viscosity /s	Filter Loss/ (mL/30 min)	Water Dissociation Ratio/%	Dynamic Shear/Pa
1	4	0.1	0.05	0.05	18.76	26.5	35.7	0.2555
2	4	0.2	0.15	0.2	23.87	19.5	14.1	0.7665
3	4	0.3	0.25	0.1	24	19.5	23.1	0.7665
4	4	0.4	0.1	0.25	24.17	29.5	37.7	0.2555
5	4	0.5	0.2	0.15	32.51	65.5	31.3	4.088
6	6	0.1	0.25	0.2	25.08	16.5	2.1	0.2555
7	6	0.2	0.1	0.1	27.57	18	0.5	0.511
8	6	0.3	0.2	0.25	33.49	16	1.1	1.533
9	6	0.4	0.05	0.15	27.40	20	1.6	0.511
10	6	0.5	0.15	0.05	34.13	19.5	1.1	0.511
11	8	0.1	0.2	0.1	24.95	16	0.5	0.7665
12	8	0.2	0.05	0.25	26.02	17.5	1.0	0.7665
13	8	0.3	0.15	0.15	49.81	16.5	1.1	0.2555
14	8	0.4	0.25	0.05	57.36	12	0.5	1.7885
15	8	0.5	0.1	0.2	41.37	17.5	1.1	0.511
16	10	0.1	0.15	0.25	46.57	15.5	0	0.511
17	10	0.2	0.25	0.15	62.62	14	0	0.511
18	10	0.3	0.1	0.05	50.95	15.5	0	0.511
19	10	0.4	0.2	0.2	59.56	14	0	0.2555
20	10	0.5	0.05	0.1	45.06	18	0	0.511
21	12	0.1	0.1	0.15	28.5	17.5	0	0.2555
22	12	0.2	0.2	0.05	120.67	14	0	0.511
23	12	0.3	0.05	0.2	57.86	18.5	0	0.511
24	12	0.4	0.15	0.1	72.45	13.5	0	0.511
25	12	0.5	0.25	0.25	55.48	13.5	0	0.511

**Table 6:** Range analysis of orthogonal test results.

Index	Factor	k					R	Optimized Combinations
		Level 1	Level 2	Level 3	Level 4	Level 5		
FV	A	24.662	29.534	39.902	52.952	66.992	42.33	A_4_B_2_C_4_D_1_
	B	28.772	52.15	43.222	48.188	41.71	23.38	
	C	35.02	34.512	45.366	54.236	44.908	19.72	
	D	56.374	38.806	40.168	41.548	37.146	19.23	
FL	A	32.1	18	15.9	15.4	15.4	16.7	A_4_B_2_C_5_D_2_
	B	18.4	16.6	17.2	17.8	26.8	10.2	
	C	20.1	19.6	16.9	25.1	15.1	10	
	D	17.5	17	26.7	17.2	18.4	9.7	
WDR	A	28.38	1.28	0.84	0	0	28.38	A_4_B_2_C_5_D_2_
	B	7.66	3.12	5.06	7.96	6.7	4.84	
	C	7.66	7.86	3.26	6.58	7.96	4.7	
	D	7.46	4.82	6.8	3.46	7.96	4.5	
DS	A	1.2264	0.6643	0.8176	0.4599	0.4599	0.77	A_4_B_2_C_5_D_2_
	B	0.4088	0.6132	0.7154	0.6643	1.2264	0.82	
	C	0.511	0.4088	0.511	1.4308	0.7665	1.02	
	D	0.7154	0.6132	1.1242	0.4599	0.7154	0.67	

## Data Availability

Data available on request due to restrictions e.g., privacy or ethical.
